# Supplementary material for: Higher Integrin Alpha 3 Beta1 Expression in Papillary Thyroid Cancer Is Associated with Worst Outcome
Source: Cancers (Basel). 2021 Jun 11;13(12):2937. doi: 10.3390/cancers13122937 (PMC8230752; doi:10.3390/cancers13122937)
Supplement: Supplementary file 1 [file cancers-13-02937-s001.zip › cancers-1246764-supplementary.pdf]

Supplementary Material

Higher Integrin Alpha 3 Beta1 Expression in Papillary Thyroid Cancer is Associated with Worst Outcome

Lorenza Mautone [1,†], Carlo Ferravante [1,2,†], Anna Tortora [1], Roberta Tarallo [1], Giorgio Giurato [1], Alessandro Weisz [1,3,*] and Mario Vitale [1,*]

Table S1. Effect of lymphocytic infiltration on integrin expression assessment.

Subunit	Pure (n=130)	LI (n=8)	p
ITGA1	2762	3158	0,575929
ITGA2	7017	7024	0,997871
ITGA3	67902	65142	0,846751
ITGA4	467	1190	0,007821
ITGA5	3622	4695	0,257799
ITGA6	5516	5785	0,801226
ITGAV	19771	16538	0,485567
ITGB1	15042	15444	0,907481
ITGAD	21	110	0,00025
ITGAL	996	2825	0,007758
ITGAM	988	2472	0,000208
ITGAX	1241	3556	0,000125
ITGB2	5397	13579	0,000245

Pure, PTC without lymphocytic infiltration; LI, PTC with 40%-15% lymphocytic infiltration; p, t-student

Table S2. Effect of stromal cells infiltration on integrin expression assessment.

Subunit	Pure (N=60)	SC (N=53)	p
ITGA1	2271	2975	0,0054
ITGA2	7908	8423	0,6712
ITGA3	73486	71338	0,6877
ITGA4	381	576	0,0378
ITGA5	3105	4421	0,0006
ITGA6	4889	5792	0,0277
ITGAV	20508	20442	0,9707
ITGB1	14418	16307	0,1835

Pure, PTC with >94% tumor cells; SC, PTC with >30% stromal cells; p, t-student
